# The observation of evolutionary interaction pattern pairs in membrane proteins

**DOI:** 10.1186/s12900-015-0033-5

**Published:** 2015-03-24

**Authors:** Steffen Grunert, Dirk Labudde

**Affiliations:** Hochschule Mittweida, University of Applied Sciences, Technikumplatz 17, Mittweida, 09648 Germany

**Keywords:** Membrane proteins, Motif, Evolutionary interaction pattern pair, EIPP, Structural similarity, Protein family affiliation

## Abstract

**Background:**

Over the last two decades, many approaches have been developed in bioinformatics that aim at one of the most promising, yet unsolved problems in modern life sciences - prediction of structural features of a protein. Such tasks addressed to transmembrane protein structures provide valuable knowledge about their three-dimensional structure. For this reason, the analysis of membrane proteins is essential in genomic and proteomic-wide investigations. Thus, many *in-silico* approaches have been utilized extensively to gain crucial advances in understanding membrane protein structures and functions.

**Results:**

It turned out that amino acid covariation within interacting sequence parts, extracted from a evolutionary sequence record of *α*-helical membrane proteins, can be used for structure prediction. In a recent study we discussed the significance of short membrane sequence motifs widely present in nature that act as stabilizing ’building blocks’ during protein folding and in retaining the three-dimensional fold. In this work, we used motif data to define evolutionary interaction pattern pairs. These were obtained from different pattern alignments and were used to evaluate which coupling mechanisms the evolution provides. It can be shown that short interaction patterns of homologous sequence records are membrane protein family-specific signatures. These signatures can provide valuable information for structure prediction and protein classification. The results indicate a good agreement with recent studies.

**Conclusions:**

Generally, it can be shown how the evolution contributes to realize covariation within discriminative interaction patterns to maintain structure and function. This points to their general importance for *α*-helical membrane protein structure formation and interaction mediation. In the process, no fundamentally energetic approaches of previous published works are considered. The low-cost rapid computational methods postulated in this work provides valuable information to classify unknown *α*-helical transmembrane proteins and to determine their structural similarity.

**Electronic supplementary material:**

The online version of this article (doi:10.1186/s12900-015-0033-5) contains supplementary material, which is available to authorized users.

## Background

Membrane proteins shape a special kind of proteins. They feature vital necessary functions in cellular processes of organisms. Fore more essential biological functions such as: photosynthesis, transport of ions and small molecules, signal transduction and light harvesting this are examples of processes which are realised by membrane proteins. The analysis of membrane proteins was shown to be an important part in the comprehension of complex biological processes in the context of proteomics and genomics [1]. Generally, membrane proteins are poorly soluble and cover a wide intra-cellular concentration range. The inaccessibility of many proteomics methods makes membrane protein analyses still an experimentally challenging field [2]. Hence, the number of known three-dimensional structures is relatively small, with 437 non-redundant membrane protein chains currently available [3-5]. Consequently, there is a necessity for approaches that allow to predict structural and functional features of unknown membrane proteins. A variety of methods have been developed to predict structural features from sequence, such as *α*-helical membrane-spanning helices and extra/intra-cellular domains (i.e. TMHMM [6,7], PHDhtm [8], MEMSAT3 [9]) as well as membrane-spanning *β*-strands of transmembrane *β*-barrel proteins (i.e. BOCTOPUS [10]). Furthermore, a major step toward *ab initio* protein structure prediction has been made through the development of new techniques for mapping energetic interactions in proteins. Here, Lockless and Ranganathan demonstrated [11] a statistical energy function as a good indicator of thermodynamic coupling in proteins. They also showed how sets of interacting residues form connected pathways in the protein fold. An existing basis for efficient energy conduction within proteins has been shown. They called their approach statistical coupling analysis (SCA) that provides the basis for further works in this area. Other approaches dealing in turn with key information to predict protein structures, which can be obtained from homologous sequences and their evolutionary variation because: “The diversity of biologic phenomena arises from the complexity and specificity of biomolecular interactions. Nucleic acid and protein polymers encode and express biologic information through the specific sequence of polymer units (residues). The sequences and corresponding molecular structures are under selective constraints in evolution [12]”.

Due to the growth of available protein sequences, many statistical methods have been developed, to compute protein three-dimensional structures from evolutionary context. Diverse contributions were involved to develop sophisticated methods to identify additional key residues that are involved in protein structure and function, especially residues that are strongly conserved within each subfamily but differ between subfamilies [13]. Previous works of Marks et al. [14,15] indicate that rich evolutionary information from genomic sequences can be efficiently mined, leading to information on evolutionary couplings between residues. Morcos et al. [16] have used information about strong constraints on their sequence variability, induced by the three-dimensional structures of homologous proteins. They developed an efficient direct-coupling analysis (DCA) [17,18] implementation to evaluate the accuracy of contact prediction for a large number of protein domains. Later on, Hopf et al. [19] presented a maximum entropy approach to infer evolutionary covariation in pairs of sequence positions of a given protein family. Generated atom models from derived pairwise distance constraints were finally used to predict the full spectrum of protein structures, functional interactions and evolutionary dynamics of unknown three-dimensional structures for 11 transmembrane proteins. A novel approach by Kamisetty et al. [20] utilizes an approximation method to obtain more accurate contact predictions for estimating residue-residue contacts in protein structures. Compared to previous methods, higher accuracy was achieved by integrating structural context and sequence co-evolution information. Hence, their method allow more accurate contact predictions from fewer homologous sequences.

Furthermore, in genome-wide membrane protein sequence analyses, numerous short conserved sequence motifs were identified [21]. These motifs support the understanding of the features that are important for establishing stability and functionality of the folded membrane protein in the membrane environment. Additionally, as addressed in [22], the analysis of sequence motifs in proteins with similar function or structure might help to identify essential functional sites and locations, which contribute to structural stability. Thus, sequence motif analysis can be helpful for numerous applications, e.g. the investigation of mutant proteins, the understanding of protein dynamics and potential effects of mutagens. During evolutionary progress the spatial structure of proteins is generally stronger conserved than the sequential amino acid composition. Adapted to the field of sequence motif analysis, structure-forming motifs point to their general importance in *α*-helical membrane protein structure formation and interaction mediation [1]. Moreover, hubs and consecutive motifs with high occurrence in certain membrane protein families can be classified as important for family-specific functional characteristics [23]. Finally, the combination of interaction information and sequence motifs with evolutionary variation can be used for three-dimensional structure prediction.

In our work we obtained key information from homologous sequences to separate and predict membrane protein structures in the context of interacting patterns and their evolutionary variation. Patterns as motif representatives are investigated regarding evolutionary covariation. Interaction information contributes to detect interacting patterns with evolutionary background. Here, we report the development of an algorithm that is involved in the extraction of interaction pattern pairs that are evolutionarily influenced. These were used for the investigation of different mutation types, which are provided by evolution to maintain structure and function. Agreeing with previous works we can state that the evolution provides basic building blocks to maintain structure and function. Related to this, family-specific interaction pattern information were used to predict unknown *α*-helical transmembrane protein structures. We have also tested our method at an already predicted structure of previous work of Hopf et al. [19]. Finally, our approach is not based on recently developed methods like SCA or DCA, but the processing of interaction and secondary structure data for predicting rich helical structure parts leads to the attachment to previous works.

## Methods

In the first step, known crystal structures of *α*-helical membrane proteins were investigated. Structural information were derived from PDBTM [24]. Currently available known *α*-helical membrane proteins were assigned to their protein families [25] using Pfam mappings. We have tested our method at two selected families with homologous sequences that contribute to generate coupling statistics (Table 1).

**Table 1:** **The analysed dataset**

**Protein Family** ^***a***^	**PDBTM** ^***b***^	**TMPad** ^***c***^	**Contacts** ^***d***^
PF01036 (Bac_rhodopsin) ^*e*^	130	102	6417
PF00230 (MIP) ^*f*^	44	40	2814

^*a*^Analysed proteins to corresponding protein family. ^*b*^Number of known structures available from PDBTM [24]. ^*c*^Number of proteins with interaction information available from TMPad [29]. ^*d*^Number of helix-helix contact information available for PDBTM assigned TMPad proteins. ^*e*^Bacteriorhodopsin-like proteins. ^*f*^Major Intrinsic Proteins.

### Evolutionary co-variations from pattern alignments (PAs)

**Figure 1:**
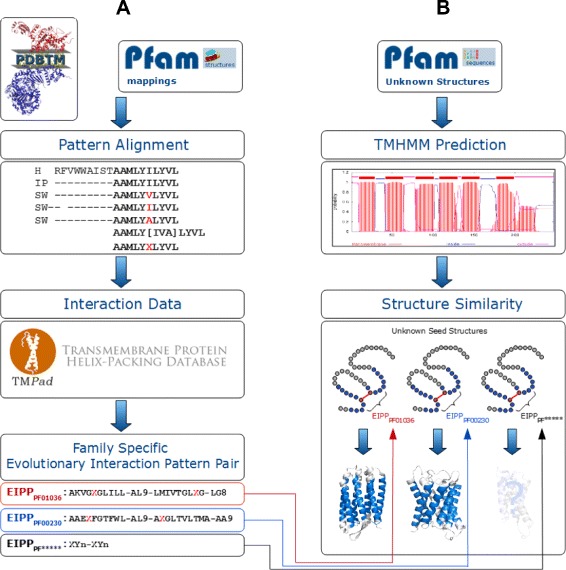
**The workflow for evolutionary interaction pattern derivation up to final structure similarity determination.** **A**: The main process to derive family-specific EIPP records. This includes the protein data aggregation from known membrane protein structures and the detection of evolutionary covariation based on pattern alignments (PAs). Together with interaction data information from TMPad [29], we obtain interacting patterns with evolutionary background, which are important for maintaining structure and function. **B**: The evaluation process includes to obtain *α*-helical sequence information from unknown membrane protein structures using by TMHMM [6,7]. Finally, signature EIPPs can be searched in unknown structures with final structure similarity determination to known structures.

Hopf et al. hypothesized and confirmed in their work [19], that the evolution conserves interactions between residues that are important to maintain structure and function. This is done by constraining the sets of mutations that are accepted at interacting sites. To find these constraint interactions within different sequence patterns, we generated PAs using a novel algorithm that detects evolutionary covariation. Aspects of this algorithm are given in this section. However, before elucidating the application of our algorithm, we want to give a short summary on the general definition of short sequence motifs, as well as the aspects of motif detection and information extraction. Consequently, the next steps are involved in motif extraction out of *α*-helical structures. Like described in previous work of [26] a motif can be written in a generalized, regular expression-like form of XYn, where X and Y correspond to amino acids separated by *n*−1 highly variable positions. For the general purpose, short sequence motifs have been extracted that contribute to build the *α*-helical structure in the transmembrane environment. Here, a naive text search algorithm was applied for motif extraction. More precisely, the algorithm mainly utilises a sliding sequence frame strategy. Beginning from the start position of the sequence, different window sizes are used to extract the underlying subsequence. Each subsequence is transcribed into its regular expression XYn. More specifically, at each sequence position *i* and *i*+*n* the algorithm returns the N-terminal residue X and the C-terminal residue Y. Note, that X and Y denote any of the 20 canonical amino acids. Redundant duplications were removed. It is known that amino acids are positioned with an average of 3.6 residues per turn in TM-helices [27] and it is also known that motifs with different length are favoured for TM-helix packing [1,28]. Based on this, the number of *n*−1 variable positions ranges within 2≤(*n*−1)≤*max*, where *max* is the maximum helix length of a protein family. Along, for a given protein each motif representative pattern was searched in all helices. If a pattern was found, the initial pattern (IP) is stored. Here, the IP represents the pattern according to which all others are aligned. To detect evolutionary covariation and to minimize the statistical noise, we have aligned patterns from other structures of the same protein-family. We ensured that these patterns, called sub-words (SWs), have up to one mutated variable position and a length of *n*_*SW*_≤*n*_*IP*_. To avoid redundancy and to minimize computational processing time, already aligned SWs were ignored. Each PA returns possible evolutionary covariation at the variable position of the aligned IP. A representative PA example is shown in Figure 1/Pattern Alignment.

### Specific evolutionary interaction pattern pairs (EIPPs)

To close the information gap when individual patterns interact with each other, we have decided to derive interaction data information from a known database. Generally, such databases allow a rapid and simple access to the required data. Helix-helix interaction information were derived from TMPad, the TransMembrane Protein Helix-Packing Database [29]. TMPad is an integrated repository of experimentally determined structural folds derived from helix-helix interactions in *α*-helical membrane proteins. Here, geometric descriptors of helix-helix interactions, topology, lipid accessibility, ligand and binding sites information are provided by TMPad. Currently, 1,107 protein entries, 4,061 protein chains and 17,413 helix-helix interactions are available. Contact information were enriched by Contacts of Structural Units (CSU) [30] derived from Weizmann Institute of Science, which provides different experimental data after the analysis of inter-atomic contacts of structural units of the protein data base (PDB) [31] entries. Now it is able to create a context between structure and helix-helix interaction information adapted to representative patterns of discriminative sequence motifs. After successfully integration of the TMPad-information to find EIPPs, helix-helix interactions were registered. An Interaction pattern pair was extracted when a contact is given only at a variable pattern position. We have ensured that at least one pattern of a given pair has mutations at the variable position. To obtain a statistical overview about the most occurring interacting motif pairs, the corresponding occurrence was recorded for each *XYn*−*XYn*. EIPPs are specific within the investigated membrane protein family. Such pairs can be considered as family-specific signatures due to their responsibility to build and stabilize the proteins structure by taking into account of the evolutionary space. Each EIPP was labelled with the corresponding protein in which the EIPP was found. Pattern interaction networks were created for final visualization and to support the understanding, how the evolution maintains attractive interaction within an EIPP. Furthermore, the existence of family-specific EIPPs was evaluated by a protein separation task. An evaluation dataset of the investigated Pfam-families PF01036 and PF00230 was derived (Table 2). Redundancy reduction was performed by assuring the family-specific number of transmembrane helices. Transmembrane helical information were obtained using TMHMM Server v. 2.0 [6,7]. Basically, TMHMM performs a prediction of intra/extra-cellular regions and integral membrane helices based on sequence. Beside per-residue predictions TMHMM also lists underlying per-residue assignment probabilities as an indicator of prediction uncertainty. TMHMM results do not always exhibit the expected typical number of 7 TM-helices (Bacteriorhodopsin-like protein) and 6 TM-helices (Major Intrinsic Proteins) in the evaluation dataset, which leads to the reduction of the evaluation dataset. Eventually, not all sequences of the evaluation dataset were included in the process. Known structure representatives were also removed.

**Table 2:** **The evaluation dataset**

**Protein Family** ^***a***^	**Proteins** ^***b***^	**Helices** ^***c***^
PF01036 (Bac_rhodopsin) ^*d*^	438	3066
PF00230 (MIP) ^*e*^	6420	38520

This dataset consists of protein family-specific representatives with unknown structures. ^*a*^Analysed proteins to corresponding protein family. ^*b*^Number of proteins available from evaluation dataset. ^*c*^Number of investigated membrane helices. ^*d*^Bacteriorhodopsin-like proteins. ^*e*^Major Intrinsic Proteins.

For the further step, protein clusters consisting of all family representative unknown structures were merged, to form a cloud and subsequently sampled. For each cloud member, family-specific EIPPs were applied on TMHMM predicted helices disregarded by mutations and under consideration of different degrees of freedom. Here, a threshold determines the number of approved variable positions within EIPPs. Matches were registered and marked in the respective helices and sequence similarity of the incurred interacting ranges compared to known structures was calculated. In addition, the family-specificity of EIPPs leads to family-specific classifiers and thus to the ability to detect an family affiliation of unknown structures that contain mutation affected homologous sequence parts. Here, it is important to mention that this task is not aimed at developing a new and better approach to classify proteins like Pfam does it with their Hidden Markov models. We will only demonstrate the specificity of mutation affected interacting sequence parts of a given protein family.

## Results and discussion

**Figure 2:**
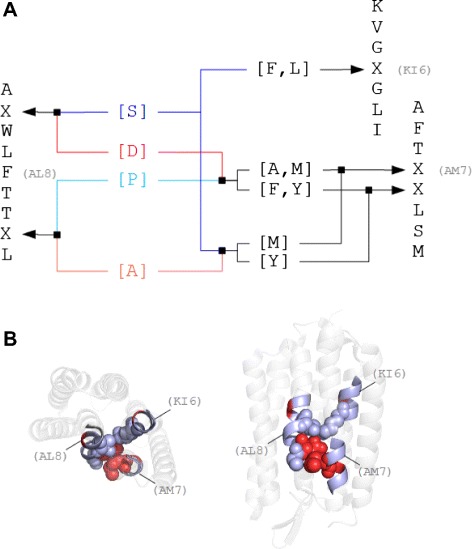
**Examples of spatially interacting sub-sequences with respect to their corresponding pattern interaction network.** **A**: More specifically, KI6 and AM7 representative patterns (right) interact with AL8 (left). All patterns have mutations. Mutational positions are marked with X. Possible amino acid replacements for AL8 representative (left) are coloured and arrows point to the respective X position. Black arrows point to the respective X position of the KI6 and AM7 representatives. With this interaction network we can track, which substitutions occur during the evolution, without influence on the interaction. **B**: The top and side view illustrations of Bacteriorhodopsin trimer (PDB-Id:1brr) are indicating where the interacting patterns are present in the helices of chain A. Generally, spheres illustrate residue-residue contacts. Red coloured spheres illustrate variable positions (X).

EIPPs were derived from known crystal structures of different membrane protein families. PAs provide evolutionarily induced variable positions within EIPPs. Like previously described, evolutionary covariation have been detected in EIPPs. In some cases, aligned SWs with up to one mutated position are responsible for multiple covariation within an EIPP member. One could have given the evolution more leeway and aligned SWs could have been designed with more than one mutated position, because it is a fact that the evolution allows more variance at the variable pattern positions to maintain structure and function. Our results show that the evolution provides basic building blocks, which are significant for the transmembrane environment like described in previous works [1,21,23]. The evolution itself determines the sequence variability and thus the variance of the variable pattern positions. If we consider each EIPP member as a basic building block we obtain a global view for this interacting sequence part in relation to a single residue. Thereby, we bypass the analysis of each residue to obtain structurally interacting units. The visualization of generated pattern interaction networks (Figure 2) supports the understanding, which pattern pairs of different length are generally involved in spatial interaction by taking into account the evolutionary background. We obtain important information about variable pattern positions that are subjected to a mutation without influencing attractive pattern interactions. The application of interaction tree schemes can lead to better indicators in laboratory mutagen investigations. More specifically, this supports the investigation of mutational variants causing different diseases like e.g. Nephrogenic diabetes insipidus.

**Figure 3:**
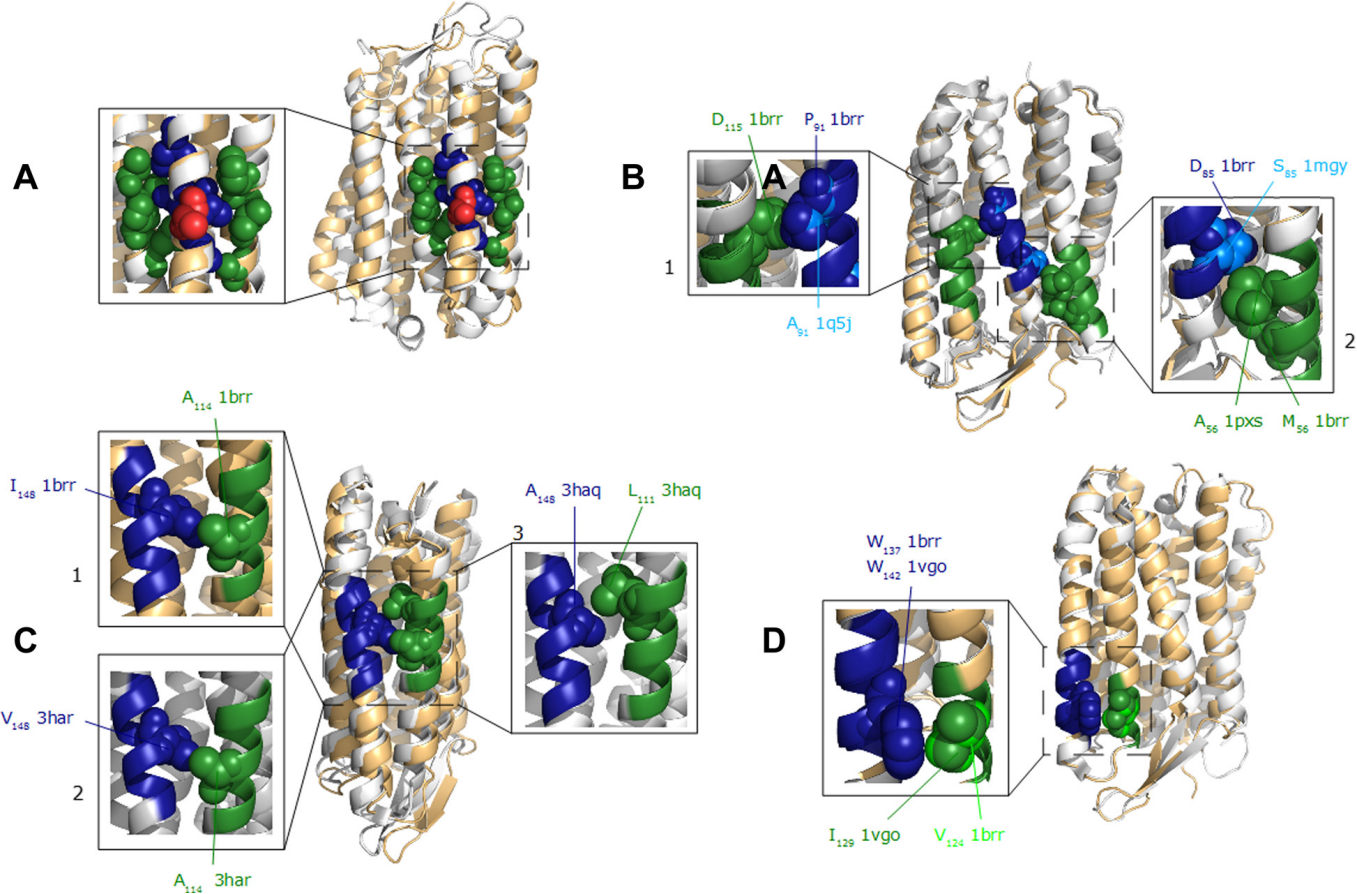
**Mutation interaction types.** Four mutation interaction types are present. Labelled spheres indicate which amino acid at specified position is present related to PDB-Id. **A**: Simple evolutionary replacements (red) around the blue and green interacting residue spheres. **B**: Interacting AL9 motifs (blue and green) with evolutionary residue substitution without loss of interaction. Mutations at one or at both interaction partner are possible. **B1**: Asp _115_ at the second position of AL9-motif pattern representative AD _115_GIMIGTL interacts with Ala _91_ or Pro _91_ of AL9-motif pattern representative A[SD] _85_WLFTT[AP] _91_LL. This is made possible by the same orientation of Ala _91_ and Pro _91_ towards its interacting counterpart. **B2**: Analogously, fourth position of AL9-motif pattern representative AFT[MA] _56_YLSMLL is designed variable with Ala _56_ or Met _56_ and interacts with Asp _85_ or Ser _85_ reason by same orientation in space. **C**: If contact information will be lost by mutation, the responsible destabilizing amino acid will be compensated by another position, in order to maintain attractive residue pair interaction [16]. **C1/C2**: Ile _148_ and Val _148_ at fifth position of AL9-motif pattern representative AMLY[VIA] _148_LYVL (blue) are able to interact with Ala _114_ at sixth position of LI8-motif representative LAL _111_ *VGA*_114_DGI (green). **C3**: Mutation with Ala _148_ causes that contact will be lost reason by to short distance to Ala _114_ counterpart. Here, Leu _111_ at third position of LI8-motif compensates the destabilizing amino acid. Evolution aims at maintaining stabilizing interactions. **D**: Trp _137/142_ is an evolutionary coupling residue which interacts with Ile _129_ or Val _124_ by full changeable residue environment around Trp _137/142_. This means that the evolutionary degree of freedom allows it to change all variable positions of an interacting pattern by keeping the conserved interaction residue.

Incidentally, for reasons of incomplete TMPad information not all position specific mutations are an integral part of our EIPPs. Only EIPP related mutations were collected if any contact could be detected from TMPad. Regarding this tree information, different known structures of PF01036 were analysed for EIPPs. The investigation of Rhodopsin-like proteins represents a major subject of research. Here different structure-function studies were performed [32,33]. Further, the investigation of active core fluctuations, the folding core and kinetics and the involved residues have been treated extensively in previous studies [34-36]. In this work, Bacteriorhodopsin-like protein structures were used to evaluate the derived EIPPs. Representatives of the statistically most interacting motifs were searched. Furthermore, long motif *XYn* (*n*=9) representative patterns show a greater tendency to interact more frequently than short ones, because of the larger number of possible residue-residue interaction combinations. The examples given in Figure 3 show, how different EIPPs comprise structural tasks and spatial interactions. Specifically, the evolution presents how EIPPs contribute to emerge different evolutionary mutation types. These types describe the sequence variability on a closer way, which has no significant influence on the protein structure and function.

These are described in more detail below:

1. Simple residue replacements that are not involved in any interaction. Tend to be an important block within an EIPP member, thus the structure can be folded without any task to build important spatial contacts (Figure 3A).
2. Contact specific mutations within evolutionary patterns. An amino acid with the responsibility to build a spatial contact to another helix will be replaced by an amino acid without modifications of the residue-residue interaction network. This can only be realized using amino acids with similar properties of the replaced residues. Here, the length and the spatial orientation play a major role to be a suitable replacement. As injunctive contact example shown in Figure 3B1: The replacement of Pro _91_ (PDB-Id: 1brr) with Ala _91_ (PDB-Id: 1q5j) within the AL9-motif representative A[DS] _85_WLFTT[PA] _91_LL has no influence to maintain the injunctive contact to their counterpart D _115_ within the AL9-motif representative AD _115_GIMIGTGL. The extended contact (Figure 3B2) between helix-helix interaction at positions 85 and 56 shows how evolutionary sequence variability contributes in such a manner that both interaction residues can be replaced by another without loosing the family-specific important contact. Here, Asp _85_ (PDB-Id: 1brr) is replaced by Ser _85_ (PDB-Id: 1mgy) within the AL9-motif representative A[DS] _85_WLFTT[PA] _91_LL. It has no influence to maintain the injunctive contacts to their counterparts Met _56_ (PDB-Id: 1brr) and Ala _56_ (PDB-Id: 1pxs) within the AL9-motif representative AFT[MA] _56_YLSMLL.
3. Morcos et al. [16] explained the simplicity between evolutionary substitutions and residue-residue contacts. “If two residues of a protein or a pair of interacting proteins form a contact, a destabilizing amino acid substitution at one position is expected to be compensated by a substitution of the other position over the evolutionary time-scale, in order for the residue pair to maintain attractive interaction”. For in-depth discussions and evaluations see [16]. These results can be seen in our frequently interacting motif pair AL8-LI8. shown in Figure 3C. C1/C2: Here, the fifth variable position of AL9-motif representative AAMLY[VAI] _148_LYVL. Val _148_ and Ile _148_ have a coupling with Ala _114_ of the LI8 representative LAL _111_*VGA*_114_DGI. C3: Mutation at position 148 with tiny Ala _148_ leads to the loss of contact to Ala _114_. Here, Leu _111_ compensates the loss of contact by interacting with tiny Ala _148_.
4. A fundamental change of variable motif positions right down to contact specific position. Thereby, common amino acids take place to cope the complete change. Such amino acids are e.g. tryptophane (Trp) with the important role in membrane proteins as described in previous work [37].

**Figure 4:**
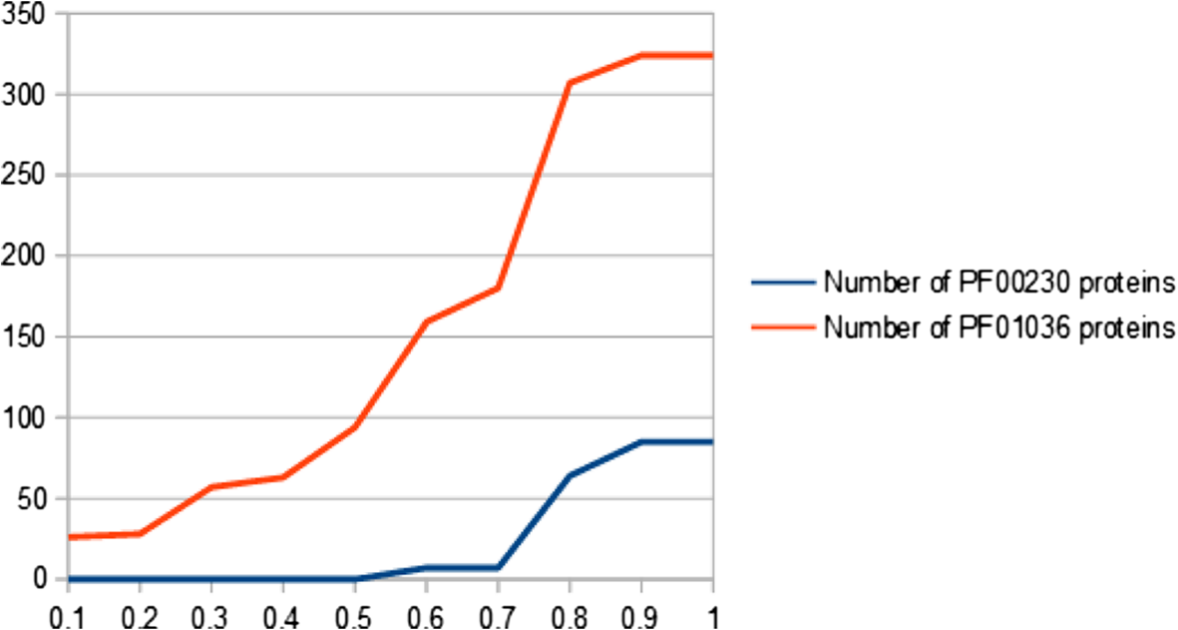
**Classification result for Bacteriorhodopsin-like (PF01036) representative unknown structures.** 372 of 438 representative proteins have been correctly assigned to PF01036. The greater the evolutionary degree of freedom (x-axis), the more variability occurs within PF01036-EIPPs. This leads to more classified proteins. On the other side, EIPPs become more unspecific for a membrane protein family which leads to wrong classified. In this case, PF01036-EIPPs were covered in 85 PF00230-proteins.

**Figure 5:**
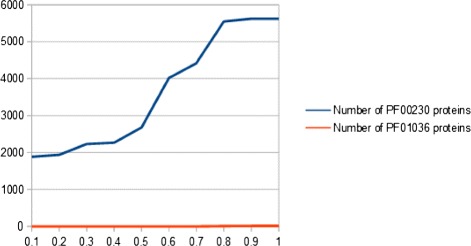
**Classification result for major intrinsic protein (PF00230) representative unknown structures.** 5,993 of 6,420 representative proteins have been correctly assigned to PF00230. The greater the evolutionary degree of freedom (x-axis), the more variability occurs within PF00230-EIPPs. This leads to more classified proteins. On the other side, EIPPs become more unspecific for a membrane protein family which leads to wrong classified. In this case, PF00230-EIPPs were covered in 14 PF01036-proteins.

In the following, a summary on how to use EIPP data for structure prediction is given. As a proof of concept, 116,810 EIPPs (PF01036) and 63,283 EIPPs (PF00230) (Table 3) were extracted from known structures of the corresponding protein families (see Additional file 1). Here, the number of EIPPs is given by interacting patterns with different lengths. These include interaction members with permanently assigned positions and members that are evolutionarily influenced. The rediscovery of EIPPs in unknown membrane protein structures of different families leads to the separation and finally to the determination of a membrane protein family affiliation. However, this is influenced by the evolutionary degree of freedom within EIPPs. With increasing variability of the variable position and under considering of the number of amino acids of a given interacting pattern, EIPPs can be recovered in other membrane protein families. That means, the greater the number of amino acids of a EIPP and the lower the evolutionary degree of freedom, the more specific is a EIPP for a membrane protein family. This has a significant impact on correctly classified proteins. In this context, the recovery of EIPPs in unknown membrane protein structures leads to the following classification results as shown in Figures 4 and 5.

**Table 3:** **Number of EIPPs derived from 130 Bacteriorhodopsin-like and 44 Major Intrinsic Protein structures**

**Variable positions**	**EIPPs**	**EIPPs**
	**PF01036**	**PF00230**
2	5754	4988
3	7656	5930
4	8784	6326
5	9864	6398
6	10382	6594
7	10302	6087
8	10529	5470
9	9692	4936
10	8727	4196
11	7797	3428
12	6545	2748
13	5538	2129
14	4569	1533
15	3498	1031
16	2530	645
17	1867	375
18	1278	218
19	801	131
20	437	68
21	187	35
22	64	12
23	8	4
24	1	1
$\sum $	116810	63283

**Figure 6:**
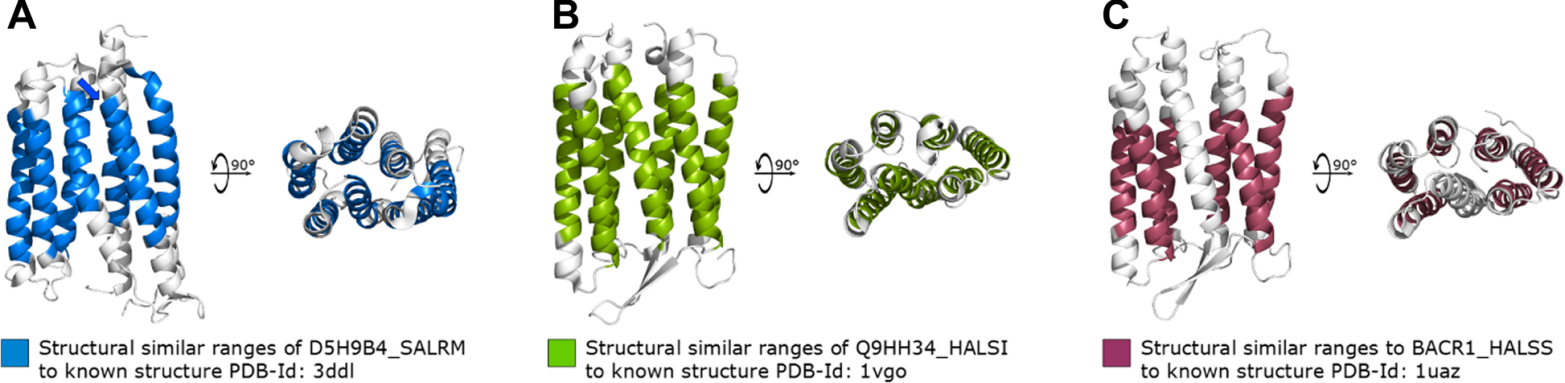
**Structural colouring of EIPP covered helical ranges with high similarity to unknown Bacteriorhodopsin-like (PF01036) structures.** Side and top-down view of the top three known structures with the highest similarity to the unknown representative. Blue, green and red coloured cartoon residue ranges are present. PF01036 family-specific EIPPs were detected in **A**: D5H9B4_SALRM **B**: Q9HH34_HALSI **C**: BACR1_HALSS and they are similar to known structures with PDB-Id **A**: 3ddl, **B**: 1vgo and **C**: 1uaz. All figures were rendered with PyMOL.

**Figure 7:**
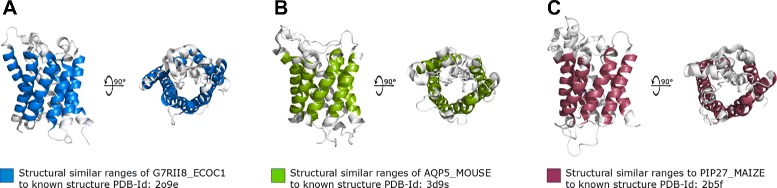
**Structural colouring of EIPP covered helical ranges with high similarity to unknown Major Intrinsic Protein (PF00230) structures.** Side and top-down view of the three known structure examples with the highest similarity to the unknown representative. Blue, green and red coloured cartoon residue ranges are present. PF00230 family-specific EIPPs were detected in **A**: G7RII8_ECOC1 **B**: AQP5_MOUSE **C**: PIP27_MAIZE and they are similar to known structures with PDB-Id **A**: 2o9e, **B**: 3d9s and **C**: 2b5f. All figures were rendered with PyMOL.

Here, 372 of 438 (PF01036) and 5,993 of 6,420 (PF00230) representative proteins have been correctly assigned to their families under the consideration of the evolutionary degree of freedom. Caused by the increase of variable positions, EIPPs became more non-specific for a membrane protein family and more proteins are incorrectly assigned. Misclassified indicate no EIPPs in the investigated membrane helices and thus no sequence similarity due to heterologous sequence parts. The reason is the restriction to allow only single mutations within aligned SWs. This leads to the fact that not all positions are considered by our algorithm. Sequence homology causes generated EIPPs to be a part of current unknown structures of the investigated protein family. Generally, our classification result shows that unknown structures can be assigned to a membrane protein family by our described method. Furthermore, registered EIPPs were marked and compared to known structures. As shown in Figures 6 and 7, the three representatives are present. These have a high structural similarity to known protein structures of the families (PF01036, PF00230). D5H9B4_SALRM, Q9HH34_HALSI and BACR1_HALSS are the top three representatives, where the most PF01036-EIPPs have been detected in TM-helices. G7RII8_ECOC1, AQP5_MOUSE and PIP27_MAIZE are three freely selected PF00230-structures with high similarity. Further similarity results are given in the attached Additional file 2.

The appropriate statistic is present in Tables 4 and 5. Considered as a whole, predicted helical ranges and finally the whole unknown structure can be compared structurally to similar known structures. For D5H9B4_SALRM this means, that 91.2% of the helical ranges be covered by PF01036-EIPPs. Followed by Q9HH34_HALSI with 90.5% and BACR1_HALSS with 85.2% structural similarity. Analogously, G7RII8_ECOC1 with 90.2%, AQP5_MOUSE with 85.2% and PIP27_MAIZE with 83.8% are covered by PF00230-EIPPs. A further evaluation has been performed. Hopf et al. have predicted [19] the unknown structures of ADR1_HUMAN with structural similarity to Bacteriorhodopsin (Pfam: PF01036, PDB-Id: 3hao) and LIVH_ECOLI with structural similarity to permease protein BtuC (Pfam: PF01032, PF00005, PDB-Id: 1l7v) in their work. We have used both structures and considered these as unknown structures. Transmembrane *α*-helical information predicted by TMHMM were applied to the classification task. ADR1_HUMAN could successfully be assigned to PF01036 and LIVH_ECOLI to PF00005. For ADR1_HUMAN this means that six of seven helices were structurally predicted with 100% similarity. The helical range of helix number 6 (H6) was covered by EIPPs with 86.4%. Besides, helical ranges of LIVH_ECOLI have high similarity to known structures of PF00005 (H1: 72.7%, H2: 50.0%, H3: 100%, H4: 90.9%, H5: 72.7%, H6: 94.1%, H7: 100%). This confirms the structure prediction result of Hopf et al. addressed to the structural similarity of ADR1_HUMAN to Bacteriorhodopsin and LIVH_ECOLI to permease protein BtuC.

**Table 4:** **Structurally similar helical ranges of unknown PF01036-structures**

	**D5H9B4_SALRM**			**Q9HH34_HALSI**			**BACR1_HALSS**	
**Helix**	**Amino acids**	**Similarity**	**Helix**	**Amino acids**	**Similarity**	**Helix**	**Amino acids**	**Similarity**
1	23	95.6%	1	23	82.6%	1	23	73.9%
2	20	95%	2	23	91.3%	2	23	78.2%
3	18	88%	3	23	95.6%	3	23	0%
4	18	100%	4	23	86.9%	4	20	90%
5	23	91.3%	5	20	100%	5	20	70%
6	23	95.6%	6	23	91.3%	6	23	69.5%
7	23	73.9%	7	23	86.9%	7	23	82.6%

For each Bacteriorhodopsin-like protein, the number of amino acids of individual TMHMM predicted helices are given. Similarity values describe consistent helical ranges, which are covered by EIPPs.

**Table 5:** **Structurally similar helical ranges of unknown PF00230-structures**

	**G7RII8_ECOC1**			**AQP5_MOUSE**			**PIP27_MAIZE**	
**Helix**	**Amino acids**	**Similarity**	**Helix**	**Amino acids**	**Similarity**	**Helix**	**Amino acids**	**Similarity**
1	23	100%	1	23	73.9%	1	23	86.9%
2	23	86.9%	2	23	73.9%	2	23	82.6%
3	23	100%	3	23	73.9%	3	23	78.2%
4	23	78.2%	4	18	94.4%	4	20	100%
5	23	78.2%	5	23	73.9%	5	23	78.2%
6	18	100%	6	18	94.4%	6	18	77.7%

For each Major Intrinsic Protein, the number amino acids of individual TMHMM predicted helices are given. Similarity values describe consistent helical ranges, which are covered by EIPPs.

Moving forward, we discuss the structural similarity results. EIPPs as interacting structural blocks are specific within a membrane protein family and for the folding of each TM-helix within a membrane protein. To recover EIPPs on a unknown structure sequence, EIPPs must occur in the helix that reflects the known structure. In this case, we had to fall back on TMHMM, a known secondary prediction tool. This dependence means that the discussed approach does not perform better than the best secondary prediction tool. On the other side, EIPPs provide TM-helical information from known structures. This leads to the possibility chance to refine secondary structure prediction tools and can be discussed in further works. Finally, our method can be used to improve sequence-based methods for classification and protein homology detection.

## Conclusion

In this work, we have demonstrated an approach for extracting short, spatially interacting amino acid sub-sequences - so called evolutionary interaction pattern pairs (EIPPs) - from known crystal structures of *α*-helical membrane protein families and underlying sequence data of protein family members. Finally, it is outlined how EIPPs can be utilized to predict protein structure. Here, covariation within motif representative homologous sequence patterns have been detected using a pattern alignment algorithm. In combination with interaction information from TMPad [29], EIPPs were obtained and employed to generate interaction trees. Thereby, we are able to show how different interacting patterns differ evolutionarily. Moreover, they have been evaluated using known structures of Bacteriorhodopsin-like proteins and discussed in detail. Here, different mutation types emerge to create an evolutionary instrument to realise sequence variability within a protein family. Furthermore, EIPPs have been used to generate family-specific classifiers. Representative proteins with unknown secondary structure have been used to predict *α*-helical sequence information using TMHMM [6,7]. Finally, family-specific protein separation has been performed and the structural similarity to known structures of the related protein family has been calculated. Addressed to structure similarity, our method describes how different interacting patterns with evolutionary background contribute to register a protein family affiliation. We are also able to determine the most similar unknown to known structures of a given *α*-helical membrane protein family. We also produced a good agreement with recently published studies that the evolution provides basic building and interacting blocks for maintaining structure and function. Due to sequence homology such blocks are repeated and we have proven structural conservation. The contemplation of a sequence from the perspective of such blocks facilitates the understanding how membrane protein structures of a family are constructed. Last but not least, low-cost rapid computational methods can be developed to support, extend or refine classification and prediction methods for membrane proteins.

## Additional files

Additional file 1
**EIPP data.** Includes derived EIPP information from families (PF00230, PF01036) with tab separated values. Can be viewed with a simple text editor. Each line consists of 7 columns: source pattern, source RegEx, destination pattern, destination RegEx, source helix, destination helix, corresponding PDB-Ids.

Additional file 2
**Similarity results.** Includes two text files for each protein family (PF00230, PF01036). Each file shows prediction results in the context of the evolutionary degree of freedom (EDF). For each protein, original and predicted helical range information are given. The end of a file shows the prediction winners.

## Abbreviations

TM: Transmembrane
SCA: Statistical coupling analysis
DCA: Direct coupling analysis
PDBTM: Protein data bank of transmembrane proteins
Pfam: The protein families database
PA(s): Pattern alignment(s)
IP(s): Initial pattern(s)
SW(s): Sub-word(s)
TMPad: Transmembrane protein helix-packing database
EIPP(s): Evolutionary interaction pattern pair(s)
CSU: Contacts of structural units
PDB: Protein data bank
SG: Steffen Grunert
DL: Dirk Labudde
